# Interrupted time series regression for the evaluation of public health interventions: a tutorial

**DOI:** 10.1093/ije/dyw098

**Published:** 2016-06-08

**Authors:** James Lopez Bernal, Steven Cummins, Antonio Gasparrini

**Affiliations:** 1 Department of Social and Environmental Health Research, London School of Hygiene and Tropical Medicine, London, UK

## Abstract

Interrupted time series (ITS) analysis is a valuable study design for evaluating the effectiveness of population-level health interventions that have been implemented at a clearly defined point in time. It is increasingly being used to evaluate the effectiveness of interventions ranging from clinical therapy to national public health legislation. Whereas the design shares many properties of regression-based approaches in other epidemiological studies, there are a range of unique features of time series data that require additional methodological considerations. In this tutorial we use a worked example to demonstrate a robust approach to ITS analysis using segmented regression. We begin by describing the design and considering when ITS is an appropriate design choice. We then discuss the essential, yet often omitted, step of proposing the impact model a priori. Subsequently, we demonstrate the approach to statistical analysis including the main segmented regression model. Finally we describe the main methodological issues associated with ITS analysis: over-dispersion of time series data, autocorrelation, adjusting for seasonal trends and controlling for time-varying confounders, and we also outline some of the more complex design adaptations that can be used to strengthen the basic ITS design.

## Introduction

Traditional epidemiological study designs such as cohort and case-control studies can provide important evidence about disease aetiology, but they are less useful as intervention studies, due to limitations such as confounding owing to group differences and, in particular, healthy user bias.^1^ Randomized controlled trials (RCTs) have long been considered the gold standard design for evaluating the effectiveness of an intervention, yet RCTs are not always possible, in particular for health policies and programmes targeted at the population level.^2–4^ Furthermore, there is often a need to retrospectively evaluate interventions which have already been implemented, often for political reasons, either without randomization or to a whole population and so without any control.^2^ The interrupted time series (ITS) study design is increasingly being used for the evaluation of public health interventions; it is particularly suited to interventions introduced at a population level over a clearly defined time period and that target population-level health outcomes.^1^^,^^5^ ITS has been used for the evaluation of a wide range of public health interventions including new vaccines, cycle helmet legislation, changes to paracetamol packaging, traffic speed zones and precautions against nosocomial infections, as well as in the evaluation of health impacts of unplanned events such as the global financial crisis.^6–11^ Other articles have outlined the design and highlighted the strengths and limitations of ITS.^1^^,^^5^^,^^12^^,^^13^ Further methodological papers have described some of the more specific in-depth modelling techniques that may be employed by those familiar with the analysis of time series data.^14^^,^^15^ Nevertheless, there is a lack of introductory guidance for those implementing an ITS evaluation for the first time. Here, we aim to demonstrate a step-by-step ITS analysis including: considering when an ITS might be an appropriate design choice and the data required; hypothesizing the type of impact the intervention will have on the outcome; how to use a regression model to analyse the effect; the main methodological issues that need to be taken into account; and finally, a brief outline of model checking techniques. A worked example is used to illustrate the methods (Box 1) and the supplementary material (available as Supplementary data at *IJE* online) includes the dataset used as well as code for use with the statistical packages Stata and R, so that readers may reproduce the analysis. 

## The interrupted time series design

**Figure 1:**
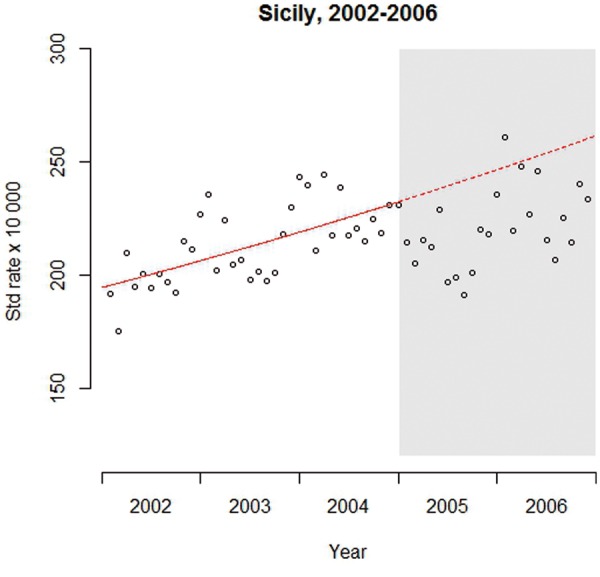
Scatter plot of example dataset. Standardized (Std) rate of ACE over time. White background, pre-intervention period; grey background, post-intervention period; continuous line, pre-intervention trend; dashed line, counterfactual scenario

A time series is a continuous sequence of observations on a population, taken repeatedly (normally at equal intervals) over time. In an ITS study, a time series of a particular outcome of interest is used to establish an underlying trend, which is ‘interrupted’ by an intervention at a known point in time. The hypothetical scenario under which the intervention had not taken place and the trend continues unchanged (that is: the ‘expected’ trend, in the absence of the intervention, given the pre-existing trend) is referred to as the ‘counterfactual’. This counterfactual scenario provides a comparison for the evaluation of the impact of the intervention by examining any change occurring in the post-intervention period.^12^^,^^17^Figure 1 illustrates the design using the smoking ban example (Box 1): the graph displays the pre-intervention trend of monthly rates of ACE admissions (continuous line), and the counterfactual scenario (dashed line). Given that most of the points lie below the counterfactual line, there is a visual suggestion of a decrease in the ACE admissions in the post-intervention period which is compatible with a possible positive impact of the smoking ban. ITS models, described below, can provide statistical evidence about whether this represents a real decrease.

### Step 1: is an interrupted time series design appropriate?

The first decision when considering an ITS is whether it is an appropriate design for the particular evaluation in question. This depends on the nature of both the intervention and the outcome of interest, as well as the type of data available:

#### The intervention

ITS requires a clear differentiation of the pre-intervention period and the post-intervention period. In some evaluations it may be difficult to define when the intervention began and to differentiate the effects of different components. This does not necessarily require the intervention to be introduced overnight but the period of implementation should be well defined so that it can be considered separately.

The implementation of the example intervention was very clear with a ban on smoking in public places throughout Italy from 10 January 2005.^16^ Where interventions have a gradual roll-out, the implementation phase can be modelled as a gradual (slope) change (see Step 2).

#### The outcome

Outcomes may take various forms such as counts, continuous data or binary variables. ITS works best with short-term outcomes that are expected to change either relatively quickly after an intervention is implemented or after a clearly defined lag.

ACEs, the outcome in the worked example, are a short-term outcome with rapid onset, and the authors of the study quote evidence suggesting that the acute effects of both active and passive smoking disappear quickly after the exposure is removed.^16^ Other diseases associated with smoking, such as lung cancer, may have been less appropriate as the timing between intervention and outcome is much less clear and can be highly variable. In this situation it may be preferable to use an intermediate outcome such as smoking prevalence.^18^

#### Data requirements

Sequential measures of the outcome should be available both before and after the intervention. There are no fixed limits regarding the number of data points, as the power depends on various other factors including distribution of data points before and after the intervention, variability within the data, strength of effect, and the presence of confounding effects such as seasonality. Zhang *et al.* conducted simulations with power calculations under different model parameters, and suggest that studies with few time points or with small expected effect sizes should be interpreted with caution as they may be underpowered, and that similar simulations should be conducted a priori under such circumstances.^19^ Power increases with the number of time points, but it is not always preferable to have more data points where historical trends have changed substantially, as this would not provide an accurate depiction of the current underlying trends.^20^ It is therefore recommended that pre-intervention data are inspected visually. Power is also increased if the numbers of data points are equally distributed before and after the intervention, though this is often not practical.^19^ Given the requirement for a relatively long time series, routine data are often most appropriate in ITS studies. As with all study designs, it is important to assess the quality of the data in terms of its validity and reliability. With routine data it is especially important to understand the potential impact of changes to data collection or recording, particularly when these coincide with the implementation of the intervention, as this could bias results.^12^

The example dataset has 59 months of routine hospital admissions data with 600-1100 ACEs at each time point. The large number of time points and minimal variability within the data provides enough power to detect relatively small changes in the hospital admission rate. In practice, as is the case in the worked example, the ITS design is often used in the evaluation of ‘natural experiments’ occurring in real-world settings and is becoming ever more possible with the increasing availability and quality of routine data spanning before and after interventions.

### Step 2: proposing the impact model

**Figure 2:**
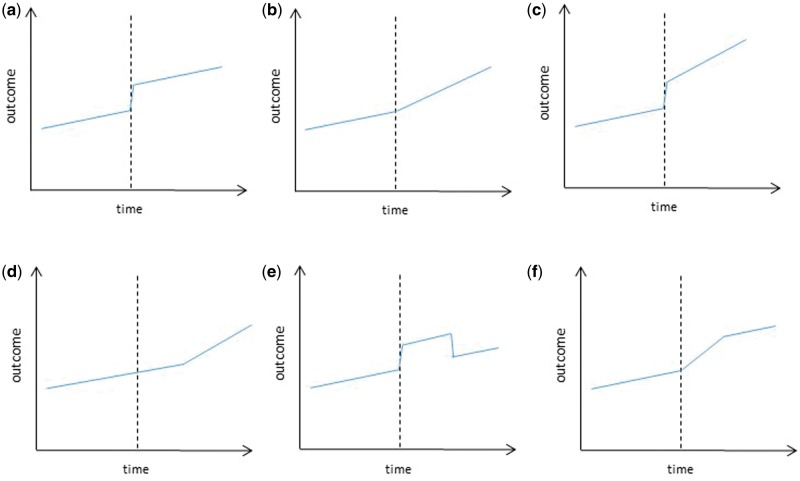
Examples of impact models used in ITS (a) Level change; (b) Slope change; (c) Level and slope change; (d) Slope change following a lag; (e) Temporary level change; (f) Temporary slope change leading to a level change.

Once an ITS design is chosen, the next step is to hypothesize how the intervention would impact on the outcome if it were effective, in particular whether the change will be a gradual change in the gradient of the trend, a change in the level or both, and whether the change will follow the intervention immediately or there will be a lag period before any effect is expected. Examples of some possible impact models are illustrated in Figure 2. It is important that this decision is made a priori based on existing literature and knowledge of the intervention and the mechanism by which it is expected to act on the outcome. Where existing knowledge of the intervention is limited, selecting the most appropriate impact model can be difficult and may require exploratory analysis of alternative data. Relying on the outcome data to select the best impact model is discouraged as this increases the likelihood of an effect being detected due to random fluctuations or chance, and consequent artefactual conclusions on the effect of the intervention.

Barone-Adesi *et al.* assumed a level change in ACEs occurring with no lag. This assumption was based on existing evidence suggesting that the acute cardiovascular risks from passive smoking disappear within a short time.^16^

### Step 3: descriptive analysis

As with all statistical analyses, initial summary statistics and plots should be undertaken to familiarize researchers with the data. This should include a scatter plot of the time series, as displayed in Figure 1, which can help to identify the underlying trend, seasonal patterns and outliers. More traditional descriptive analyses, such as summaries and bivariate comparisons between the outcome and potential time-varying confounders, as well as simple before-and-after comparisons, are recommended.

### Step 4: regression analysis

A minimum of three variables are required for an ITS analysis:

- T: the time elapsed since the start of the study in with the unit representing the frequency with which observations are taken (e.g. month or year);
- $X_{t\;}$: a dummy variable indicating the pre-intervention period (coded 0) or the post-intervention period (coded 1);
- $Y_{t}:$ the outcome at time t.

In standard ITS analyses, the following segmented regression model is used:

$$Y_{t}=\beta_{0\;}+\beta_{1}T+\beta_{2}X_{t\;}+\;\beta_{3}TX_{t\;}$$

where $\beta_{0\;}$represents the baseline level at T = 0, $\beta_{1}$ is interpreted as the change in outcome associated with a time unit increase (representing the underlying pre-intervention trend), $\beta_{2\;}$is the level change following the intervention and $\beta_{3}$ indicates the slope change following the intervention (using the interaction between time and intervention: $TX_{t\;}$). The regression model above represents the impact model (c) in Figure 2; models (a) and (b) can easily be specified by excluding the terms $\beta_{3}TX_{t\;}$ or $\beta_{2\;}X_{t\;}$, respectively. Impact models (d)-(f) require slightly more complex variable specifications (Supplementary Appendix 5, available as Supplementary data at *IJE* online).

**Figure 3:**
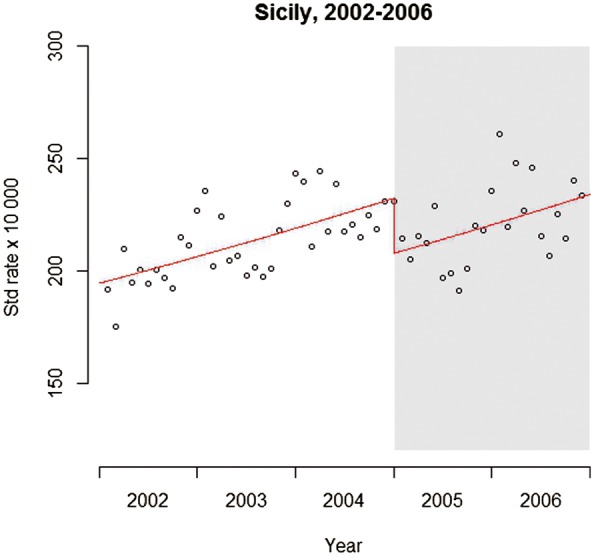
Interrupted time series with level change regression model. Line: predicted trend based on the unadjusted regression model

In our example, T,X and Y are shown in Table 1. As is frequently the case in population health evaluations, here the outcome is a count and, without loss of generality, a Poisson regression model was used. Other regression models can equally be used, such as ordinary least squares (linear) regression for continuous outcomes, for example the duration of cycling trips in an ITS study looking at the impact of public transport strikes on usage of a bicycle share programme in London.^21^ Most of the steps described in this tutorial remain the same for the analysis of other types of outcomes, unless specifically stated. Furthermore, the age-standardized population (in person-years) was included as an offset variable to convert the outcome into a rate and adjust for any potential changes in the population over time (though this is not essential if the population is relatively stable over time, as in this case). Given that a level change model was hypothesized, the interaction term for the slope change is not required in the model. This model, shown using Stata code and R code in Supplementary Appendices 2 and 3 (available as Supplementary data at *IJE* online), suggests that there is very strong evidence of a reduction in ACEs following the smoking ban, with a decrease of 11% [relative risk (RR) 0.894; 95% confidence interval (CI) 0.864-0.925; *P* < 0.001) as illustrated in Figure 3.

**Table 1.** Excerpt from the example dataset

Year	Month	Time elapsed	**Smoking ban**^a^	ACEs	Std popn
		**(**T)	**(**X)	(Y)	
2004	1	25	0	914	381656.3
2004	2	26	0	808	383680
2004	3	27	0	937	383504.2
2004	4	28	0	840	386462.9
2004	5	29	0	916	383783.1
2004	6	30	0	828	380836.8
2004	7	31	0	845	383483
2004	8	32	0	818	380906.2
2004	9	33	0	860	382926.8
2004	10	34	0	839	384052.4
2004	11	35	0	887	384449.6
2004	12	36	0	886	383428.4
2005	1	37	1	831	388153.2
2005	2	38	1	796	388373.2
2005	3	39	1	833	386470.1
2005	4	40	1	820	386033.2
2005	5	41	1	877	383686.4
2005	6	42	1	758	385509.3
2005	7	43	1	767	385901.9
2005	8	44	1	738	386516.6
2005	9	45	1	781	388436.5
2005	10	46	1	843	383255.2
2005	11	47	1	850	390148.7
2005	12	48	1	908	385874.9

ACEs, hospital admissions for acute coronary event; Std popn, age-standardized population in person-years.^16^

a Smoking ban: 0, smoking ban not in place; 1, smoking ban in place.

### Step 5: addressing methodological issues

Whereas the basic model implemented so far provides an indication of the potential association between the intervention and the outcome, there are a number of distinctive issues with time series data that may need to be addressed in order to improve the robustness of the analysis.

#### Seasonality

**Figure 4:**
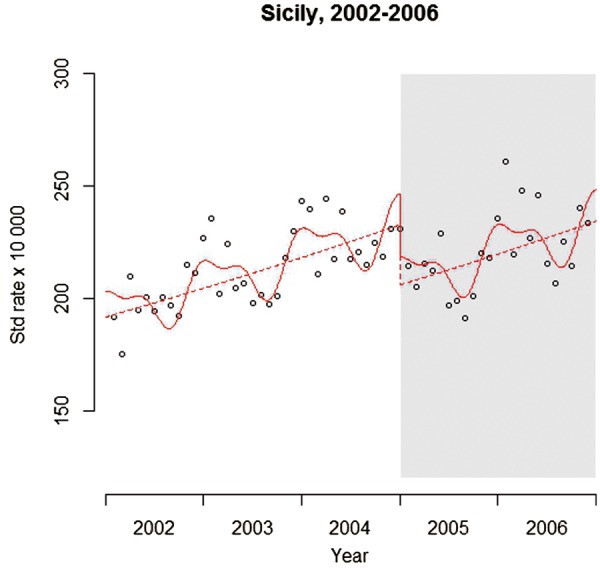
Model adjusted for seasonality. Solid line: predicted trend based on the seasonally adjusted regression model. Dashed line: de-seasonalized trend

Many diseases and other outcomes have a seasonal pattern and this is evident in the ACE data in Figure 1. Seasonality can cause two problems: first, if there is an uneven distribution of months before and after the intervention, such as a higher proportion of winter months, this could bias the results, especially in the analysis of short series. Second, outcomes in one month tend to be more similar to those in neighbouring months within the same time of year, leading to autocorrelation and over-dispersion (discussed below). There are a range of methods for controlling for seasonality and other long-term trends; these include: a model stratified by the calendar month (or other time period); or using more complex functions such as Fourier terms (pairs of sine and cosine functions); or splines. Each of these methods is explained in more detail by Bhaskaran *et al.* 2013.^22^Figure 4 shows the example analysis after adjustment for seasonality through a Fourier term, with results suggesting that the association is largely unaffected: (RR: 0.885; 95% CI 0.839-0.933; *P* < 0.001).

#### Time-varying confounders

One of the strengths of ITS studies is that they are generally unaffected by typical confounding variables which remain fairly constant, such as population age distribution or socioeconomic status, as these only change relatively slowly over time and are normally taken into account when modelling the underlying long-term trend. Nevertheless, ITS can be affected by time-varying confounders that change more rapidly. Seasonality can be considered a time-varying confounder; others may include levels of a particular infectious disease that is prone to outbreaks, weather events etc. Where such time-varying confounders have been measured, they can be controlled for by including variables representing them in the regression model, as is commonly undertaken in other epidemiological analyses. A special category of time-varying confounders are other events that occur around the same time as the intervention and that potentially influence the outcome. These might include other simultaneous interventions targeting the same outcome, or risk factors for that outcome, or natural events that could affect the outcome.

Potential time-varying confounders in the smoking ban study might include changes in diagnostic procedures for detecting ACEs, for example a new troponin test had been progressively implemented in Italy since 2000,^23^ or interventions targeting other risk factors for cardiovascular disease such as a healthy eating intervention.

#### Use of controls and other more complex ITS designs

Where time-varying confounders are either unmeasured or unknown, a range of design adaptations can be used to control for possible concurrent events including: adding a control group or control outcome which would not have been affected by the intervention (known as a controlled interrupted time series); using a multiple baseline design whereby the intervention is introduced in different locations at different times; or adding additional phases so that the intervention is first introduced and then withdrawn to establish whether withdrawal of the intervention leads to a reversal of the effect. These methods are described in more detail elsewhere.^12^^,^^13^^,^^15^^,^^24^

#### Over-dispersion

An assumption of the Poisson distribution is that the variance is equal to the expected count. However, in analyses of real data, the variance frequently tends to be greater (a phenomenon known as over-dispersion) which would lead to incorrect estimation of the standard errors. A scaling adjustment is therefore made to correct to the model to correct this, detailed by Bhaskaran *et al*. and illustrated in Supplementary Appendices 2 and 3 (available as Supplementary data at *IJE* online).^22^ This issue does not apply for the analysis of continuous outcomes when a Gaussian distribution, including a residual error to be estimated, is assumed. In the example this widens the 95% confidence interval marginally to 0.839-0.953, yet there is still very strong evidence of an effect (*P* = 0.001).

#### Autocorrelation

A second assumption of standard regression models is that observations are independent. This assumption is often violated in time series data because consecutive observations tend to be more similar to one another than those that are further apart, a phenomenon known as autocorrelation. Fortunately, in many epidemiological data, autocorrelation is largely explained by other variables, in particular seasonality (discussed above); therefore, after controlling for these factors, residual autocorrelation is rarely a problem. Nevertheless, autocorrelation should always be assessed by examining the plot of residuals and the partial autocorrelation function and, where data are normally distributed, conducting tests such as the Breusch-Godfrey test.^22^^,^^25^ Where residual autocorrelation remains, this should be adjusted for using methods such as Prais regression or autoregressive integrated moving average (ARIMA), described in more detail elsewhere.^26^^,^^27^ There is very little evidence of autocorrelation in the worked example and even less after adjustment for seasonality (Supplementary Appendices 2 and 3, available as Supplementary data at *IJE* online).

#### Further extensions

Further extensions, as described by Bhaskaran *et al*. (2013) for environmental time series, and in more detail elsewhere, can also be applied to ITS studies, including: stratified analyses according to potential effect-modifying variables; increasing power by allowing different locations to have trends modelled individually rather than relying on the aggregated trend; and modelling non-linear trends.^9^^,^^22^^,^^23^

### Step 6: model-checking and sensitivity analyses

A range of model-checking techniques have been described above including plotting residuals and partial autocorrelation functions. Furthermore, sensitivity analyses can be conducted to test the impact of varying a range of model assumptions, such as different lags, types of impact model or approaches to adjusting for seasonality.^11^^,^^22^

## Summary

In this article we have introduced the key steps for readers undertaking an ITS study, including highlighting the main methodological considerations and how they may be addressed. ITS analyses are one of the strongest evaluative designs when randomization is not possible; furthermore, they often allow a more detailed assessment of the longitudinal impact of an intervention than may be possible with an RCT and, given that they are frequently undertaken in real-world settings, may have stronger external validity. Another important feature in ensuring that research gets translated into practice is that graphical and numerical presentation of results can be easily understood by those with little expert knowledge of statistical and epidemiological methods. Nevertheless, there are some important threats to the validity of ITS analyses, perhaps the most important of which include the potential for the erroneous conclusion of intervention effectiveness due to data-driven model specification, and lack of control for time-varying confounders. It is therefore essential that any interpretations regarding the causal effect of any association are undertaken with caution and that some of the key steps to analysis highlighted in this article, such as a priori model specification and methodological extensions to control for confounders, are followed. With carefully planned analyses and handling of potential threats to validity, ITS can provide valuable evidence about the effectiveness of health interventions.

## Supplementary Data

Supplementary data are available at *IJE* online.

## Supplementary Material

Supplementary Data
