# Corrigendum to: Interrupted time series regression for the evaluation of public health interventions: a tutorial

**DOI:** 10.1093/ije/dyaa118

**Published:** 2020-09-03

**Authors:** James Lopez Bernal, Steven Cummins, Antonio Gasparrini

First published online: 8 June 2016, *Int J Epidemiol* 2017; 46: 348–55. doi: https://doi.org/10.1093/ije/dyw098

The originally published version of this article contained an algebraic definition of the regression model for interrupted time series (ITS) that could lead to erroneous interpretations of the estimated parameters. This model was presented in the equation at page 351, right column, and the following text. We provide here a more accurate definition.

The correct equation should have been:

$$Y_{t}=\beta_{0}+\beta_{1}T+\beta_{2}X_{t}+\beta_{3}\left(T-T_{0}\right)\cdot X_{t}$$

The following text below the equation at page 351 (right column) and page 352 (left column), should read:

“where $\beta_{0}$ represents the baseline level, $\beta_{1}$ is interpreted as the change in outcome associated with a time unit increase (representing the underlying pre-intervention trend), $\beta_{2}$ is the level change following the intervention, and $\beta_{3}$ indicates the slope change following the intervention (with $T_{0}$ as the time of the beginning of the intervention). The regression model above represents the impact model (c) in Figure 2; models (a) and (b) can easily be specified by excluding the terms $\beta_{3}\left(T-T_{0}\right)$ or $\beta_{2}X_{t}$, respectively. Impact models (d)-(f) require slightly more complex variable specifications (Supplementary Appendix 4, available as Supplementary data at IJE online).”.

The R code in Supplementary Appendix 3 has been fixed accordingly, while the Stata code in Supplementary Appendix 2 was already consistent with the definition provided here.

Finally, the Supplementary Material did not include a Supplementary Appendix 5 referred to in the text, which provides additional details on ITS model specifications. This is now included as Supplementary Appendix 4.

## Supplementary Material

dyaa118_Supplementary_Data

