# Supplementary material for: Corrigendum to: Interrupted time series regression for the evaluation of public health interventions: a tutorial
Source: Int J Epidemiol. 2020 Sep 3;50(3):1045. doi: 10.1093/ije/dyaa118 (PMC7750921; doi:10.1093/ije/dyaa118)
Supplement: dyaa118_Supplementary_Data [file dyaa118_supplementary_data.zip › Web_Appendix_4.docx]

## Web appendix 4: Further model specifications

### Slope change following a lag (figure 2 (d)):

Here, the start of the intervention can be coded as 1 following the lag (rather than immediately after the intervention). For example, in Table 4.1, if we assume a 6 month lag and the intervention was introduced in January 2005, we code the intervention as 1 from July 2005. We then use the usual slope change regression model: $Y_{t}=\beta_{0}+\beta_{1}T+ \beta_{3}\left( T-T_{0} \right)\cdot X_{t}$ with $\beta_{3}$representing the slope change.

Table 4.1: variable specification for slope change following a lag

| Year | Month | Time elapsed | Smoking ban lagged | ACEs | Std popn |
| --- | --- | --- | --- | --- | --- |
|  |  | ($T)$ | ($X)$ | $(Y)$ |  |
| 2004 | 1 | 25 | 0 | 914 | 381656.3 |
| 2004 | 2 | 26 | 0 | 808 | 383680 |
| 2004 | 3 | 27 | 0 | 937 | 383504.2 |
| 2004 | 4 | 28 | 0 | 840 | 386462.9 |
| 2004 | 5 | 29 | 0 | 916 | 383783.1 |
| 2004 | 6 | 30 | 0 | 828 | 380836.8 |
| 2004 | 7 | 31 | 0 | 845 | 383483 |
| 2004 | 8 | 32 | 0 | 818 | 380906.2 |
| 2004 | 9 | 33 | 0 | 860 | 382926.8 |
| 2004 | 10 | 34 | 0 | 839 | 384052.4 |
| 2004 | 11 | 35 | 0 | 887 | 384449.6 |
| 2004 | 12 | 36 | 0 | 886 | 383428.4 |
| 2005 | 1 | 37 | 0 | 831 | 388153.2 |
| 2005 | 2 | 38 | 0 | 796 | 388373.2 |
| 2005 | 3 | 39 | 0 | 833 | 386470.1 |
| 2005 | 4 | 40 | 0 | 820 | 386033.2 |
| 2005 | 5 | 41 | 0 | 877 | 383686.4 |
| 2005 | 6 | 42 | 0 | 758 | 385509.3 |
| 2005 | 7 | 43 | 1 | 767 | 385901.9 |
| 2005 | 8 | 44 | 1 | 738 | 386516.6 |
| 2005 | 9 | 45 | 1 | 781 | 388436.5 |
| 2005 | 10 | 46 | 1 | 843 | 383255.2 |
| 2005 | 11 | 47 | 1 | 850 | 390148.7 |
| 2005 | 12 | 48 | 1 | 908 | 385874.9 |

### Temporary level change (figure 2 (e)):

This model may be used where a reversible intervention is introduced temporarily. For example, if the intervention were introduced for six months, then withdrawn, the variables may be specified as per Table 4.2. A level change regression model could then be run as follows: $Y_{t}=\beta_{0}+\beta_{1}T+\beta_{2}X_{t}+ \beta_{3}W_{t}$ where $\beta_{2}$represents the level change at introduction and $\beta_{3}$represents the remaining level change at withdrawal. Under the assumption that the latter is null, and that the level comes back to the underlying trend, $\beta_{3}W_{t}$can be excluded.

Table 4.2: variable specification for a temporary level change

| Year | Month | Time elapsed | Smoking ban introduced | Smoking ban withdrawn | ACEs | Std popn |
| --- | --- | --- | --- | --- | --- | --- |
|  |  | ($T)$ | ($X)$ | (W) | $(Y)$ |  |
| 2004 | 1 | 25 | 0 | 0 | 914 | 381656.3 |
| 2004 | 2 | 26 | 0 | 0 | 808 | 383680 |
| 2004 | 3 | 27 | 0 | 0 | 937 | 383504.2 |
| 2004 | 4 | 28 | 0 | 0 | 840 | 386462.9 |
| 2004 | 5 | 29 | 0 | 0 | 916 | 383783.1 |
| 2004 | 6 | 30 | 0 | 0 | 828 | 380836.8 |
| 2004 | 7 | 31 | 0 | 0 | 845 | 383483 |
| 2004 | 8 | 32 | 0 | 0 | 818 | 380906.2 |
| 2004 | 9 | 33 | 0 | 0 | 860 | 382926.8 |
| 2004 | 10 | 34 | 0 | 0 | 839 | 384052.4 |
| 2004 | 11 | 35 | 0 | 0 | 887 | 384449.6 |
| 2004 | 12 | 36 | 0 | 0 | 886 | 383428.4 |
| 2005 | 1 | 37 | 1 | 0 | 831 | 388153.2 |
| 2005 | 2 | 38 | 1 | 0 | 796 | 388373.2 |
| 2005 | 3 | 39 | 1 | 0 | 833 | 386470.1 |
| 2005 | 4 | 40 | 1 | 0 | 820 | 386033.2 |
| 2005 | 5 | 41 | 1 | 0 | 877 | 383686.4 |
| 2005 | 6 | 42 | 1 | 0 | 758 | 385509.3 |
| 2005 | 7 | 43 | 0 | 1 | 767 | 385901.9 |
| 2005 | 8 | 44 | 0 | 1 | 738 | 386516.6 |
| 2005 | 9 | 45 | 0 | 1 | 781 | 388436.5 |
| 2005 | 10 | 46 | 0 | 1 | 843 | 383255.2 |
| 2005 | 11 | 47 | 0 | 1 | 850 | 390148.7 |
| 2005 | 12 | 48 | 0 | 1 | 908 | 385874.9 |

## Temporary slope change leading to a level change (Figure 2(f)):

This model could be used to represent a phase during which an intervention was gradually introduced in which case we may be interested in the slope change as the intervention was phased in, as well as the absolute level change following its introduction. The variable specification in Table 4.3 can be used with the following regression model: $Y_{t}=\beta_{0}+\beta_{1}T+\beta_{2}X_{t}$. The parameterization of $X_{t}$in Table 4.3 allows a gradual change change over 5 months, and $\beta_{2}$represents the full change at the end of the implementation period.

Table 4.3: variable specification for a temporary level change or temporary slope change

| Year | Month | Time elapsed | Intervention phased in | ACEs | Std popn |
| --- | --- | --- | --- | --- | --- |
|  |  | ($T)$ | ($X)$ | $(Y)$ |  |
| 2004 | 1 | 25 | 0 | 914 | 381656.3 |
| 2004 | 2 | 26 | 0 | 808 | 383680 |
| 2004 | 3 | 27 | 0 | 937 | 383504.2 |
| 2004 | 4 | 28 | 0 | 840 | 386462.9 |
| 2004 | 5 | 29 | 0 | 916 | 383783.1 |
| 2004 | 6 | 30 | 0 | 828 | 380836.8 |
| 2004 | 7 | 31 | 0 | 845 | 383483 |
| 2004 | 8 | 32 | 0 | 818 | 380906.2 |
| 2004 | 9 | 33 | 0 | 860 | 382926.8 |
| 2004 | 10 | 34 | 0 | 839 | 384052.4 |
| 2004 | 11 | 35 | 0 | 887 | 384449.6 |
| 2004 | 12 | 36 | 0 | 886 | 383428.4 |
| 2005 | 1 | 37 | 0.2 | 831 | 388153.2 |
| 2005 | 2 | 38 | 0.4 | 796 | 388373.2 |
| 2005 | 3 | 39 | 0.6 | 833 | 386470.1 |
| 2005 | 4 | 40 | 0.8 | 820 | 386033.2 |
| 2005 | 5 | 41 | 1 | 877 | 383686.4 |
| 2005 | 6 | 42 | 1 | 758 | 385509.3 |
| 2005 | 7 | 43 | 1 | 767 | 385901.9 |
| 2005 | 8 | 44 | 1 | 738 | 386516.6 |
| 2005 | 9 | 45 | 1 | 781 | 388436.5 |
| 2005 | 10 | 46 | 1 | 843 | 383255.2 |
| 2005 | 11 | 47 | 1 | 850 | 390148.7 |
| 2005 | 12 | 48 | 1 | 908 | 385874.9 |
